# Data on the environmental sustainability index of large Brazilian companies

**DOI:** 10.1016/j.dib.2019.103819

**Published:** 2019-03-09

**Authors:** F.S. Rosa, R.J. Lunkes, M.M.B. Brizzolla

**Affiliations:** a Federal University of Santa Catarina (UFSC), SC, Brazil; b Northwestern Regional University of the State of Rio Grande do Sul (UNIJUI), RS, Brazil

## Abstract

This data article is related to the research article “Exploring the Relationship between Internal Pressures, Greenhouse Gas Management and Performance of Brazilian Companies” (Rosa et al, 2019). This presents data collected from 63 large Brazilian companies organized as follows: Industrial Goods (2); Construction and Transportation (12); Cyclical Consumption (3); Non-Cyclical Consumption (3); Financial and Other (6); Basic Materials (4); Information Technology (2); Telecommunications (2); and Electricity (29). These data were collected by the questionnaire administered by the São Paulo Stock Exchange (Brazil) about internal factors of Corporate Social Responsibility (CSR) of an Environmental Management System (EMS) and the reduction of emission of greenhouse gases and economic performance. The data were used to show a relationship between the use of EMS and the reduction of greenhouse gas emissions and economic performance. The data also show that risk management and the pursuit of opportunities related to climate change and establishing incentives at different levels linked to reduction targets, together with employee awareness and training, have an impact on managing greenhouse gas emissions and the greenhouse effect.

**Table:** Specifications table

*Subject area*	*Applied Social Sciences*
*More specific subject area*	*Business, Management and Accounting (General)*
*Type of data*	*Table, figure*
*How data was acquired*	*The primary data were collected in a questionnaire administered by the São Paulo Stock Exchange (BOVESPA) to companies from different economic sectors and business portfolios that are publicly from 63 Brazilian companies listed on the stock exchange and ISE in 2016.*
*Data format*	*Analysed*
*Experimental factors*	*To examine how internal CSR pressures moderate the relationship between EMS and environmental and economic performance, we consider nine variables. As the independent variable, we use two variables to represent the EMS, one related to the mitigation of impacts, and the other related to the prediction of impacts. We use four dependent variables, two of which represent RGHG aspects and two, EP. Finally, three moderating variables are used to measure the internal CSR pressures: accountability, incentives, and training.*
*Experimental features*	*Data characteristics such as minimum sample size, abnormal data, and scale of measurement (i.e., the use of different scale types) are among the most often stated reasons for applying PLS-SEM.*
*Data source location*	*Brazil*
*Data accessibility*	*Data within this article are acquired from companies listed on the São Paulo Stock Exchange, ISE and B3 under the corporate sustainability aspect, based on economic efficiency, environmental balance, social justice and corporate governance. Site:*http://iseb3.com.br/questionario-ise-b3-2016---versao-final
*Related research article*	*Rosa, F.S., Lunkes, R.J., Brizzolla, M.M.B, 2019. Exploring the Relationship between Internal Pressures, Greenhouse Gas Management and the Performance of Brazilian Companies. J. Clean. Prod. (in press).*[1]

**Table:** 

**Value of the data** • The data are used to show the complex relationships that exist between the internal factors of the CSR, the implementation of EMS to reduce greenhouse gas emissions and economic performance. Specifically, the data suggest that internal factors related to managers and employees are important in the effective use of EMS for reducing greenhouse gas emissions and for economic performance. • This study also extends the knowledge about the subject, demonstrating that the use of EMS, aligned with internal factors of CSR, will produce better results.

## 1 Data

The primary data were collected in a questionnaire administered by the São Paulo Stock Exchange (BOVESPA) to companies from different economic sectors and business portfolios that are publicly available from 63 Brazilian companies listed on the stock exchange and ISE in 2016.

The Corporate Sustainability Index (ISE) is an investment portfolio that has sustainable criteria for the selection of companies that make up the company. The ISE is a tool for comparative analysis of the performance of companies listed in B3 under the corporate sustainability aspect based on economic efficiency, environmental balance, social justice and corporate governance. There are three categories of ISE participation: Eligible, Training and Simulation. Participation in the Training and Simulation categories is only for the purpose of preparing companies for the future participation of the ISE investment portfolio. The Eligible category is formed by the 200 companies with the most liquid shares during the year. Companies interested in participating in the process are enrolled electronically and receive access to the system online. This questionnaire is prepared by the B3 team in partnership with FGV (Fundação Getúlio Vargas) and is updated every year. To be included in the index, companies must agree to respond. This questionnaire is composed of seven dimensions: General; Nature of the Product; Corporate governance; Economic-financial; Environmental; Social; and Climate changes. Each of the dimensions is composed of criteria, and each criterion has different weights. Weights are defined mainly by the relevance of the theme in the current context of corporate management and the demands of society. In addition, each criterion is composed of indicators that are calculated from a set of questions, according to Table 1.

**Table 1:** Comm: Commitments; Ali: Alignment; Strat: Strategic Perspective; Ethics and Transp: Ethics and Transparency; Personal Impacts of Product Use; Diffuse Impacts of Product Use; Compliance: Legal compliance; Property: Property; Admin Council: Administrative Council; Manag: Management; Audit: Audit and inspection; conflict of interest: Conduct and conflict of interest; Politic:s Politics; Manag: Management; Perform: Performance; Compliance: Legal compliance; Politics: Politics; Manag: Management; Perform: Performance; Compliance: Legal compliance; Politics : Politics; Manag : Management; Perform: Performance; Compliance: Legal compliance; Politics: Politics; Manag : Management; Perform: Performance; Report : Report.

Dimension	Criteria	Weight	Indicator/questions
General	Commitments	15	1. Fundamental commitment (7 questions) 2. Voluntary commitments (8 questions)
	Alignment	33	3. Consistency of commitments (8 questions) 4. Engagement with stakeholders (3 questions) 5. Performance and recognition (5 questions)
	Strategic Perspective	17	6. Strategy and positioning (6 questions) 7. Value chain (6 questions)
	Ethics and Transparency	35	8. Defense of competition (2 questions) 9. Prevention and fight against corruption (6 questions) 10. Political action (4 questions) 11. Reports (10 questions)
Nature of Product	Personal Impacts of Product Use	30	1. Risks to the consumer or third parties (7 questions)
	Diffuse Impacts of Product Use	60	2. Diffuse risks (15 questions) 3. Observance of the precautionary principle (3 questions)
	Legal compliance	10	4. Consumer information (2 questions) 5. Judicial or administrative sanctions (4 questions)
Corporate governance	Property	30	1. Relationships between partners (13 questions) 2. Transparency (2 questions) 3. Legal compliance (3 questions) 4. Governance of subsidiaries, affiliates and/or subsidiaries (2 questions)
	Administrative Council	30	5. Structure of the board of directors (6 questions) 6. Dynamics of the board of directors (5 questions)
	Management	10	7. Quality of management (5 questions)
	Audit and inspection	10	8. Accountability (9 questions)
	Conduct and conflict of interest	20	9. Conduct and conflict of interest (11 questions)
Economic-financial	Politics	20	1. Corporate strategy and risk (4 questions)
	Management	40	2. Corporate risks and opportunities (10 questions) 3. Crisis and contingency plan (3 questions) 4. Intangible assets (1 questions) 5. Performance management (1 question)
	Performance	30	6. Financial statements (3 questions) 7. Economic profit (2 questions) 8. Balance of growth (1 question)
	Legal compliance	10	9. History (3 questions)
Environmental	Politics	*	This dimension has criteria that change according to the business segment of the company, since each segment has a specific environmental impact.
	Management		
	Performance		
	Legal compliance		
Social	Politics	24	1. Commitment to fundamental principles and rights in labor relations (6 questions) 2. Commitment to the community (6 questions) 3. Respect for privacy, use of information and marketing (3 questions)
	Management	40	4. Implementation of commitments with principles and fundamental rights in labor relations (8 questions) 5. Relationship with the community (11 questions) 6. Relationship with customers and consumers (10 questions)
	Performance	26	7. Diversity and equity (16 questions) 8. Supplier management (4 questions) 9. Resolution of customer and consumer demands (5 questions)
	Legal compliance	10	10. Internal public (2 questions) 11. Customers and consumers (4 questions) 12. Society (6 questions)
Climate change	Politics	17,25	1. Commitment, comprehensiveness and dissemination (7 questions)
	Management	46,75	2. Responsibility (4 questions) 3. Mitigation management (7 questions) 4. Management of adaptation (3 questions) 5. Management systems (2 questions)
	Performance	20	6. Results (2 questions)
	Report	16	7. Disclosure (2 questions)

Adapted from ISE (2018).

In addition to the responses to the questionnaire, the selection process also includes an analysis of the documents submitted by the companies to substantiate the information provided, and for the final deliberation conducted by the ISE Deliberative Council, the CISE. The technical management of this process is conducted by the FGV, with the assurance of KPMG. At the end of the selection process, up to 40 companies are selected to compose the index of the corresponding evaluation year.

## 2 Experimental design, materials and methods

The questionnaire administered by the São Paulo Stock Exchange is a tool used for a comparative analysis of the performance of companies listed in B3 regarding corporate sustainability. The companies voluntarily participate in this of the portfolio of investments denominated Corporate Sustainability Index (ISE). The questionnaire is developed by the FGV team and is formed of seven dimensions that evaluate different aspects of sustainability: Overall Dimension; Product Nature Dimension; Corporate Governance Dimension; Economic, Financial, Environmental and Social Dimensions; and Climate Change Dimension. All the data can be found on the website of the São Paulo Stock Exchange (http://iseb3.com.br/questionario-ise-b3-2016---versao-final). In this study, only the issues related to greenhouse gases were analysed. It was intended to collect specific data on (a) Environmental Management Systems - Mitigation and Prediction of impacts; (b) Internal Corporate Social Responsibility pressures - CSR-Accountability, CSR-Incentives and CSR-Training; (c) Environmental Performance - Establishment and Reduction of Greenhouse Gas targets; and (d) Economic Performance - ROA and ROE. The descriptive statistics of the analysed variables of the 63 companies is presented in Table 2.

**Table 2:** Descriptive statistics of the 63 large companies of Brazil.

Variable	Item	n	Mean	Median	Min	Max	STDVE
Mitigation of impacts	CLI10	63	2.10	2.00	0.00	3.00	0.79
Adapting to climate change	CLI14	63	2.79	4.00	0.00	4.00	1.51
Emission reduction	CLI18	63	2.48	3.00	0.00	6.00	1.93
Emission reduction targets	CLI19	63	1.29	2.00	−1.00	2.00	0.93
Return on assets	ROA	63	423.51	217.00	−474.00	1526.00	448.95
Return on equity	ROE	63	1650.37	925.00	−4238.00	6988.00	2070.99
Accountability	CLI5	63	2.78	3.00	0.00	4.00	1.28
Incentives	CLI6	63	1.71	2.00	−1.00	4.00	2.08
Training	CLI7	63	6.83	7.00	4.00	9.00	1.49

### 2.1 Variable measurement

To examine how internal pressures from CSR moderate the relationship between EMS and environmental and economic performance, this study uses nine variables. As an independent variable, we use two variables that represent the EMS [2], [3], [4], [5], [6], one related to the mitigation of impacts and the other related to the prediction of impacts. We use four variables as dependent variables, two of which represent GGR (Greenhouse Gas Reduction) aspects [7], [8] and two of which represent economic performance (EP) [6], [7], [8], [9], [10], [11], [12], [13], [14]. Finally, three moderating variables are used to measure the CRS Internal Pressures [5], [12], [1]: Accountability, Incentives and Training.

The model variables are thus measured:

(a) Environmental Management System: measured by two aspects: Mitigation Impacts (Scale from 0 (no emissions) to 3 (accomplishes emissions mitigation and uses compensation measures), and Prediction of impacts conducted studies in the last 3 years).
(b) Internal Corporate Social Responsibility pressure: measured by three aspects: CSR-Accountability (Scale from 0 (no accountability) to 4 (all hierarchical levels are accountable for risks and opportunities), CSR-Incentives 5 (all hierarchical levels are included in the incentives plan), and CSR-Training (Scale from 0 (none) to 8 (promotes training to employees and other workers/stakeholders);
(c) Environmental Performance, measured by two aspects: Proof RGHG (Scale from 0 (no inventory) to 6 (has inventory of direct and indirect emissions throughout the production process and value chain) to 2 (attained and/or exceeded);
(d) Economic Performance - ROA and ROE.

### 2.2 Empirical model

To measure the level of EMA, CSR and EP were used as ordinal scales ranging from 0 to 8, with “0” representing the worst level of response, in which there is an absence of data or inferior performance.

In the present study, it is understood that EMS enables the reduction of GG emissions and the improvement in economic business performance. In the analysis of the data, it is considered that the internal pressures of the CSR influence environmental and economic performance. Thus, it is considered that the aspects related to the responsibility and incentives of managers and the training of stakeholders and employees influence the relationship between EMS and environmental and economic performance. The internal pressures of CSR moderate the relationship between EMS and RGG and between EMS and EP.
