# Supplementary material for: Data on the environmental sustainability index of large Brazilian companies
Source: Data Brief. 2019 Mar 9;24:103819. doi: 10.1016/j.dib.2019.103819 (PMC6535814; doi:10.1016/j.dib.2019.103819)
Supplement: Supplementary file 1 — Multimedia Component 1 [file mmc1.docx]

Declarations of interest: none

**Data on the Environmental Sustainability Index of Large Brazilian Companies.** ROSA, F. S. ^a^, LUNKES, R. J. ^b^,BRIZZOLLA, M. M. B.^c^
